# Psychological distress and its risk factors during COVID-19 pandemic in Saudi Arabia: a cross-sectional study

**DOI:** 10.1186/s43045-021-00089-6

**Published:** 2021-02-08

**Authors:** Ghada Moh Samir Elhessewi, Fatmah Almoayad, Samira Mahboub, Anwar Mohammed Alhashem, Lamiaa Fiala

**Affiliations:** 1 Department of Health Sciences, College of Health and Rehabilitation Sciences, Princess Nourah Bint Abdulrahman University, P.O. Box 84428, Riyadh, 11671 Kingdom of Saudi Arabia; 2 Nursing Administration Department, Damanhour University, Damanhour, Egypt; 3 Department of Public Health, Community, Environmental and Occupational Medicine, Faculty of Medicine, Suez Canal University, Ismailia, Egypt

**Keywords:** Psychological distress, COVID-19, Saudi Arabia, K10

## Abstract

**Background:**

The control measures during COVID-19 such as curfew, lockdown, and social distancing had observed differences in controlling the spread of the disease around the Kingdom of Saudi Arabia; however, they might contribute to psychological illnesses such as anxiety, depression, panic disorder, and distress. A cross-sectional descriptive study was conducted to assess psychological distress and the factors affecting it among general population in the Kingdom of Saudi Arabia during the COVID-19 pandemic.

**Results:**

Seven hundred and thirty-nine people completed an online questionnaire which included the Kessler Psychological Distress Scale (K10). Psychological distress was found among 35% of the study sample. Western and northern regions reported higher rates of distress. In addition, higher rates were found among younger and unmarried individuals. Distress was significantly positively correlated with perceptions of susceptibility and severity of COVID-19 infection, and the fear to lose a job as a result of the pandemic and the related precautions.

**Conclusion:**

Psychological support programs should be provided targeting high-risk groups of younger and unmarried population. Further research should be conducted to evaluate the effectiveness of psychological support interventions.

## Background

The Saudi Ministry of Health announced the first case of COVID-19 on 2 March 2020 [24], after 12 weeks from the spread of the pandemic in sixty-six countries around the globe [5]. Up to 2 July 2020, the total number of cities reporting at least one case was 197 and a total number of deaths up to 1698 cases across the Kingdom. Makkah region has the most cases (57,548 cases) than the capital region Riyadh which had a total of 52,936 cases [23]. In only 1 month (March 2020), the Saudi authorities had restricted sports events; closed educational facilities, parks, and malls; and suspended domestic public transportation, and had started partial curfew [29, 32].

The control measures such as curfew, lockdown, and social distancing had observed differences in controlling the spread of the disease around the Kingdom [32]; however, they contribute to psychological illnesses such as anxiety, depression, panic disorder, and distress [1, 12, 26]. The prevalence of some mental conditions varies from one country to another. In China, in a study of 52,730 participants, psychological distress affected almost 35% of their participants [26]. Similarly, a couple of reviews in Italy found an extremely high range level of depression among 15.4% of Italians, 11.5% extremely anxious, 12.6% extremely stressed [21], 57% with sleeping problems, and 41.8% suffers from high distress [10]. In previous studies, women, elderly, and young are more vulnerable to develop stress and suffer from post-traumatic syndrome after pandemics [21, 26, 28], and it is reported that excessive exposure to news, social media, and TV influences by exuberating stress [1, 26]. The psychological effect of COVID-19 has been also reported in Saudi Arabia. Different ranges of distress were reported among 40% of the general Saudi population as a result of COVID-19 as reported by Al-Hanawi et al. [2]. Moreover, Alkhamees et al. [3] reported moderate to severe psychological impact among 23.6% of the Saudi general public. Both previously mentioned studies found higher rates among females, young people, and health practitioners [2, 3]. While literature established the psychological impact of the pandemic on Saudi population, there was limited discussion of the factors influencing it. Yet, Alkhamees et al. [3] found that commitment to infection control preventive measures is negatively significantly associated with stress and anxiety levels. The psychological distress is a reaction to a stressor that can cause timely or permanent dysfunction [27]. Its combined symptoms such as depression, anxiety, difficulty to sleep, and lethargy, and all may vary from person to person [17]. The person may experience coping difficulty, fluctuations of emotions, and feeling irritable, mostly [27].

The Saudi authorities noted the rise of psychological disorder and several health messages and guidelines distributed to the public. For example, Saudi CDC [11] provided a preventive guide for mental and social health focused on prevention and stress and fear management during the pandemic. They targeted the general population and how to take care of children and the elderly as well as tips for healthcare providers and managers of health facilities. The National Centre for Mental Health Promotion [25] promoted free call counseling center with mental health specialist for help during the pandemic as well as provided some health messages on psychological tips during lockdown such as how to increase resilience and to deal with loneliness and isolation especially with elderly and with special need family members. Some hospitals act by educating the public about mental health, such as King Faisal Specialist Hospital and Research Center had provided social media messages on how to manage stress and anxiety during the pandemic [16].

The aim of this study was to explore the psychological distress and the factors affecting it among residents of Saudi Arabia during the pandemic COVID-19. By understanding the current situation of psychological distress in Saudi Arabia, the health practitioner can develop rapid protection and intervention programs that enhance mental health, especially among the distressed population.

## Methods

### Design of the study

This is a cross-sectional descriptive study to assess psychological distress and the factors affecting it among general population in the Kingdom of Saudi Arabia (KSA) during the COVID-19 pandemic. Data was compiled during June 2020.

### Participants of the study

The study population included those of 18 years and older, of any nationality, and are present inside KSA during the pandemic. Non-probability, convenient sampling technique was used to recruit the participants. The research questionnaire was available through Microsoft forms and distributed via social media in both Arabic and English languages. With a confidence level of 99% and total population in KSA of 25.8 million [13], the sample size was 666 participants, calculated using the sample size calculator the Survey System [30]. The questionnaire was completed by 760 participants of which 739 met the inclusion criteria.

### Research instrument

The research tool used for this research consisted of 37 questions distributed into 3 sections as follows:

- Section 1: consisted of 8 questions which covered the sociodemographic characteristics of the participants including age, gender, income, education, marital status, employment status, region of residence, and housing condition.
- Section 2: Kessler Psychological Distress Scale (K10) was used to assess the psychological distress among the participants [14]. The K10 scale included 10 questions which measure emotional distress by assessing anxiety and depressive symptoms and has been recommended for use among the general population. It uses a 5-point scale ranging from “None of the time” which is assigned a score of 1 to “All of the time” which is assigned a score of 5. The maximum total score is 50, and the minimum is 10, and the results indicate the likelihood of the mental health as follows: well < 20, have a mild mental disorder 20–24, have moderate mental disorder 25–29, and have a severe mental disorder ≥ 30 [4].
- Section 3: targeted factors related to COVID-19 pandemic that may influence the psychological status. It included four questions to assess perceived threat of COVID-19, five questions for the perceived seriousness of the COVID-19 infection, four questions for the commitment to precautionary measures, and four questions assessing the perception of effectiveness of those precautionary measures. Finally, participants were asked about their fear of losing their jobs as a result of the pandemic lockdown, the feasibility of staying home and not going out unless necessary, and the feasibility of avoiding social gatherings with family and friends. This section was assessed using a 5-point Likert scale ranging from “strongly agree” which was assigned a score of five to “strongly disagree” which was assigned a score of one.

### Validity and reliability

The tool used for this research was developed after extensive review of the literature. K10 tool which was used to assess psychological distress is a valid and reliable tool that has been used in different contexts [8, 31]. The tool was translated to Arabic using forward-backward technique and displayed to participants in both Arabic and English. Reliability test was done using Cronbach’s α test which resulted in *α* =0.89 for the Arabic translation of K10. Validity was tested for all questions and was all significantly correlated with the overall score *r* > 0.5. Reliability test for the influencing factors scored as follows: perceived threat *α* = 0.7, for perceived seriousness *α* = 0.8, for precautionary measures *α* = 0.7, and for the perception of effectiveness of preventive measures *α* = 0.7.

### Data analysis

Data was analyzed using the statistical software (JMP version 14.2). Frequency tables were used for descriptive data. Associations between categorical variables were done using the Chi-square test. Correlation between scale variables was assessed using Pearson correlation coefficient test. Multiple linear regression was performed to predict K10 score among the studied sample. Statistical significance was set at *p* ≤ 0.05 and high significance at *p* < 0.01.

## Results

The sociodemographic characteristics of the study population are presented in Table 1. More than half of the sample were females (68%), and nearly one fourth were young adults aged less than 35 years old. The participants in this study had a good educational level as 82% had at least a college degree, and most of them were married (76%). Regarding working status, 58% were working, and 15% were retired. More than half of the participants were from the Central Region of KSA (62%), one fourth from the Western Region, and those from other regions were about 14% of the whole sample.

**Table 1:** Sociodemographic characteristics of the study sample (*n* = 739)

Sociodemographic characteristics	***N***	%
**Sex**		
Female	507	68.8
Male	230	31.2
**Age (years)**		
19 to 35 years	211	28.7
36 to 50 years	307	41.8
More than 50 years	217	29.5
**Marital status**		
Married	563	76.2
Not married	176	23.8
**Education**		
Less than college degree	129	17.5
College degree or postgraduate	610	82.5
**Working status**		
Working	435	58.9
Not working	186	25.2
Retired	118	15.9
**Residence in KSA**		
East	51	6.9
Central	463	62.7
North	20	2.7
South	32	4.3
West	173	23.4
**Nationality**		
Saudi	649	87.2
Non-Saudi	90	12.8
**Income (SR**^a^ **per month)**		
Less than 8000	232	31.4
8000–16,000	251	33.9
More than 16,000	256	34.7

^a^Saudi riyal

The overall prevalence of psychological distress of different degrees was 35% (*n* = 260), and only about 65% (*n* = 479) were likely to be well. The highest prevalence of those who were likely to be well were living in the Eastern Region (66.6%, *n* = 34) and the Central Region (67.39%, *n* = 312), while the lowest prevalence was in the Northern region (only 45%, *n* = 9). Regarding prevalence of different degrees of psychological disorders, Northern Region demonstrated the highest prevalence of mild disorder (30%, *n* = 6), and one fifth (*n* = 7) of those living in the Southern Region suffered from moderate disorder. The overall prevalence of severe disorder was 8% (*n* = 60) where the Western and the Northern Regions had the highest prevalence of 10% each (*n* = 18, 2 respectively).

During the past month, nearly 40% of the participants felt nervous (*n* = 298) and restless (*n* = 288) and about 30% felt that everything was an effort (*n* = 230). About one fifth of the studied population felt depressed and so sad that nothing could cheer them up at least sometimes in the past month (*n* = 177).

Table 2 illustrates the association between psychological distress and social factors among the population living in KSA. Older age groups demonstrated better psychological status. Those who were more than 50 years old had a significant higher prevalence of normal psychological status compared to younger age groups of 36 to 50 years and those of less than 36 years (76%, 63%, and 55% respectively, *p* < 0.01). Those who were 19 to 35 years old had a significantly higher prevalence of severe psychological disorder than older groups (12% vs 8% and 2% respectively). There was also a significant association between being unmarried and having psychological distress. Those who were unmarried had a significantly higher prevalence of severe psychological disorder than married (13% vs 6%, *p*<0.01).

**Table 2:** Association between psychological distress and social factors among the population living in KSA

Social factors	Psychological status				***p*** value
	Likely to be well	Mild disorder	Moderate disorder	Severe disorder	
**Age**					
19–35 years	117 (55.45%)	40 (18.96%)	27 (12.80%)	27 (12.80%)	0.0001^†^
36–50 years	196 (63.84%)	49 (15.96%)	35 (11.40%)	27 (8.79%)	
More than 50 years	165 (76.04%)	33 (15.21%)	13 (5.99%)	6 (2.76%)	
**Nationality**					
Saudi	421 (64.87%)	105 (16.18%)	70 (10.79%)	53 (8.17%)	0.3
Non-Saudi, Arabic speakers	49 (62.03%)	17 (21.52%)	6 (7.59%)	7 (8.86%)	
Non-Saudi, non-Arabic speakers	9 (81.82%)	2 (18.18%)	0 (0%)	0 (0%)	
**Marital status**					
Unmarried	97 (55.11%)	34 (19.32%)	21 (11.93%)	24 (13.64%)	0.003^†^
Married	382 (67.85%)	90 (15.99%)	55 (9.77%)	36 (6.39%)	
**Gender**					
Female	319 (62.92%)	86 (16.96%)	62 (12.23%)	40 (7.89%)	0.08
Male	158 (68.70%)	38 (16.52%)	14 (6.09%)	20 (8.70%)	
**Income (SR**^a^ [**per month)**					
Less than 8000	147 (63.36%)	37 (15.95%)	27 (11.64%)	21 (9.05%)	0.4
8000–16,000	153 (60.96%)	49 (19.52%)	27 (10.76%)	22 (8.76%)	
More than 16,000	179 (69.92%)	38 (14.84%)	22 (8.59%)	17 (6.64%)	
**Working status**					
Not working or retired	199 (65.46%)	49 (16.12%)	33 (10.86%)	23 (7.57%)	0.9
Working	280 (64.37%)	75 (17.24%)	43 (9.89%)	37 (8.51%)	
**Being a health care worker**					
No	412 (66.03%)	102 (16.35%)	63 (10.10%)	47 (7.53%)	0.3
Yes	67 (58.26%)	22 (19.13%)	13 (11.30%)	13 (11.30%)	

*p < 0.05

^†^*p* < 0.01

^a^Saudi riyal

No significant association was found between psychological status and all of the following social factors: nationality, gender, income, working status, and being a health care worker (*p* > 0.05).

Table 3 demonstrates the correlation between K10 score of the psychological distress and factors related to the COVID-19 pandemic among the population living in KSA. It was found that there was a positive significant correlation between the K10 score of psychological distress and the scores of perceived seriousness of the disease, perceived threat of the COVID-19 infection, fear of losing job because of reasons related to the pandemic, and difficulty of staying home and not going out unless necessary. (*p* < 0.01 for all of them except difficulty of staying home that had *p* < 0.05.) There was also a highly significant negative correlation between K10 score of psychological distress and the scores of feasibility of avoiding social gathering, commitment to all preventive measures, and the perception of effectiveness of theses preventive measures (*p* < 0.01).

**Table 3:** Correlation between K10 score of psychological distress and factors related to COVID-19 pandemic among the population living in KSA

Factors	K10 score
Perceived seriousness of the disease	*r* = 0.14^†^
Perceived threat of infection	*r* = 0.13^†^
Fear of losing job because of reasons related to the pandemic	*r* = 0.18^†^
Difficulty of staying home and not going out unless necessary	*r* = 0.10*
Feasibility of avoiding social gatherings with family and friends	*r* = − 0.11^†^
Commitment to all preventive measures	*r* = − 0.14^†^
Perception of effectiveness of preventive measures	*r* = − 0.17^†^

*p < 0.05

^†^*p* < 0.01

All the nine factors that showed significant association with psychological distress were entered a regression model to predict psychological distress among population in KSA (Table 4). It was found that five factors can successfully predict the psychological disorder. These factors are age, perceived seriousness of the disease, perception of effectiveness of precautionary measures, fear of losing job because of reasons related to the pandemic, and difficulty of staying home and not going out unless necessary (*P* < 0.001 for all of them and for the whole model).

**Table 4:** Multiple linear regression predicting psychological distress among the studied sample

Term	Estimate	Std error	***t*** ratio	Prob > |***t***|
Intercept	25.091142	2.8948	8.67	< .0001*
Age	− 0.124101	0.021613	− 5.74	< .0001*
Marital status [unmarried]	0.351404	0.319698	1.10	0.2721
Perceived threat of infection	0.0577026	0.089452	0.65	0.5191
Perceived seriousness of the disease	0.2796199	0.071428	3.91	< .0001*
Perception of effectiveness of preventive measures	− 0.29369	0.09266	− 3.17	0.0016*
Fear of losing job because of reasons related to the pandemic	0.7454765	0.227063	3.28	0.0011*
Difficulty of staying home and not going out unless necessary	0.5926451	0.214181	2.77	0.0058*
Commitment to preventive measures	− 0.189432	0.157251	− 1.20	0.2287
Feasibility of avoiding social gatherings with family and friends	− 0.26727	0.38231	− 0.70	0.4847

Dependent variable: psychological distress

*P < 0.01

## Discussion

The results have revealed that more than one third the study population suffer different degrees of psychological distress, and around two thirds were likely to be well. Psychological distress was significantly higher among younger age groups and unmarried individuals. There was also a significant positive correlation between psychological distress and perceptions of susceptibility and severity, and the fear to lose a job as a result of the pandemic and the related precautions.

K10 tool, which was used to assess psychological distress in the current study, is expected to find psychological distress in around 13% of general population and in 25% clinical setting ([4]; Ronald C [14]). However, in this study, 35% of the people have scored 20 or more which reflects a degree of distress. While these results are higher than what is usually expected in general population, it is not surprising as psychological health is known to be affected by pandemics [9]. Moccia et al. [22] used the same tool to assess the psychological health burden of the pandemic in Italy and found that distress is recorded in 38% of their study sample. In Saudi Arabia, a study was conducted by Alkhamees et al. [3] during an early stage of the pandemic to assess its psychological impact. The study reported that nearly quarter of the study sample suffered moderate to severe psychological impact because of the pandemic. The increase in numbers found in the current study which is conducted at a later stage of the pandemic is expected as suggested by Bonanno et al. [7] who studied the longitudinal psychological effect of SARS epidemic in Hong Kong. Additionally, Al-Hanawi et al. [2], who conducted a study during the month of May indicated that 40% of the general public in Saudi Arabia suffer from psychological distress caused by COVID-19. Thus, as COVID-19 lasts, more people are expected to be affected by its psychological impact, and more efforts are needed for psychological support.

A closer look into the results reveals that severe distress existed in 8.1% of the sample. This is in line with the findings of Al-Hanawi et al. [2] who reported severe distress in 7% of their study sample, while even higher rates were reported by Alkhamees et al. [3] as symptoms of severe stress were found in 13.7%, symptoms of severe anxiety in 13.9%, and symptoms of severe depression in 16.4%. Moreover, moderate psychological distress was found in 10.3% of the study sample which although it may not be as serious as severe distress, yet literature suggests it still requires intervention and support (Ronald C [15].). In fact, these are alarming results that require well-planned intervention as according to a systematic review conducted to assess the impact of psychological distress on health, all studies reported a negative effect on health [6].

To provide the appropriate intervention, the target population should be identified. In the current study, age had significant negative correlation with psychological distress. People who were in the age group 19–35 years old scored highest in severe psychological distress and lowest in normal rates (12%, 55% respectively), people between 36 to 50 years old scored 8% in severe distress and 63% in normal rates, and finally, those who were older than 50 years old showed the lowest rates of severe distress and the highest of normal rates (2%, 76% respectively). Additionally, severe psychological distress was significantly higher in unmarried individuals. This supports other national studies where tendency to have psychological distress and adverse mental health was higher among younger adults [2, 3]. Several explanations were offered for the distress among younger ages including the access to vast amount of information via social media and the stronger effect of lockdown on younger people [2].

It was found that there was a positive significant correlation between the K10 score of psychological distress and the scores of perceived seriousness of the disease, perceived threat of the infection, fear of losing job because of reasons related to the pandemic, and difficulty of staying home and not going out unless necessary. This result seems normal that people are more prone to anxiety, depression, and fear when confronting unfamiliar diseases, as the number of confirmed cases and deaths from COVID-19 increase, the public’s psychological state is likely to worsen [20]. This result is in line with previous literature that confirms that certain level of psychological distress could induce people to be more committed to control measures in order to reduce the speed of respiratory infection transmission [19]. Additionally, Alkhamees et al. [3] have confirmed the negative association between commitment to infection control measures and both stress and anxiety during COVID-19 in Saudi Arabia.

### Strengths and limitations

It is a strength of this study that it was conducted during the pandemic so it could provide an accurate reflection of people’s experience. Additionally, the tool used for data collection is highly reliable. The weakness of the study is using convenient sampling method. However, the use of online surveys has been considered as an efficient method in research as it is an effective way of reaching study population and saves time and cost [18].

## Conclusion

To conclude, psychological distress was found among one third of the study sample during the pandemic, which is higher than the expected general population anxiety rates. This was significantly apparent in younger age groups and unmarried people. Based on the findings of this study, we recommend for psychological support programs to target high-risk population mainly younger and unmarried population. Further research should be conducted to evaluate the effectiveness of psychological support interventions.

## Data Availability

The datasets used and/or analyzed during the current study are available from the corresponding author on reasonable request.

## Abbreviations

K10: Kessler Psychological Distress Scale
KSA: Kingdom of Saudi Arabia

## References

[1] Ahmed MZ, Ahmed O, Aibao Z, Hanbin S, Siyu L, Ahmad A (2020). Epidemic of COVID-19 in China and associated Psychological Problems. Asian J Psychiatr.

[2] Al-Hanawi MK, Mwale ML, Alshareef N, Qattan AM, Angawi K, Almubark R (2020). Psychological distress amongst health workers and the general public during the COVID-19 pandemic in Saudi Arabia. Risk Manage Healthcare Policy.

[3] Alkhamees AA, Alrashed SA, Alzunaydi AA, Almohimeed AS, Aljohani MS (2020). The psychological impact of COVID-19 pandemic on the general population of Saudi Arabia. Compr Psychiatry.

[4] Andrews G, Slade T (2001). Interpreting scores on the Kessler psychological distress scale (K10). Aust N Z J Public Health.

[5] ARAB-NEWS (2020). Saudi Arabia announces first case of coronavirus.

[6] Barry V, Stout ME, Lynch ME, Mattis S, Tran DQ, Antun A (2020). The effect of psychological distress on health outcomes: a systematic review and meta-analysis of prospective studies. J Health Psychol.

[7] Bonanno GA, Ho SMY, Chan JCK, Kwong RSY, Cheung CKY, Wong CPY (2008). Psychological resilience and dysfunction among hospitalized survivors of the SARS epidemic in Hong Kong: a latent class approach. Health Psychol.

[8] Bougie E, Arim RG, Kohen DE, Findlay LC (2016). Validation of the 10-item Kessler psychological distress scale (K10) in the 2012 Aboriginal peoples survey. [report]. Health Rep.

[9] Brooks SK, Webster RK, Smith LE, Woodland L, Wessely S, Greenberg N (2020). The psychological impact of quarantine and how to reduce it: rapid review of the evidence. Lancet.

[10] Casagrande M, Favieri F, Tambelli R, Forte G (2020). The enemy who sealed the world: effects quarantine due to the COVID-19 on sleep quality, anxiety, and psychological distress in the Italian population. Sleep Med.

[11] CDC (2020) Saudi Center for Disease prevention and control. Novel corona virus (2019-nCoV) infection guidelines V1.2. Saudi Center for Disease Prevention and Control Ministry of Health, Riyadh

[12] Galea S, Merchant RM, Lurie N (2020). The mental health consequences of COVID-19 and physical distancing: the need for prevention and early intervention. JAMA Intern Med.

[13] GASTAT (2019). Statistical mid year estimate https://www.stats.gov.sa/sites/default/files/population_by_age_groups_and_gender_ar.pdf. Accessed 15th June 2020.

[14] Kessler RC, Andrews G, Colpe LJ, Hiripi E, Mroczek DK, Normand S-L (2002). Short screening scales to monitor population prevalences and trends in non-specific psychological distress. Psychol Med.

[15] Kessler RC, Green JG, Gruber MJ, Sampson NA, Bromet E, Cuitan M (2010). Screening for serious mental illness in the general population with the K6 screening scale: results from the WHO world mental health (WMH) survey initiative. Int J Methods Psychiatr Res.

[16] KFSHRC (2020). Managing stress & anxiety. https://www.kfshrc.edu.sa//en/home/covid/managingstress. Accessed 13 July 2020.

[17] LAbate, L. (2012). *Mental illnesses: understanding, prediction and control*: BoD–Books on Demand.

[18] Lefever S, Dal M, Matthiasdottir A (2007). Online data collection in academic research: advantages and limitations. Br J Educ Technol.

[19] Leung G, Lam T, Ho L, Ho S, Chan B, Wong I (2003). The impact of community psychological responses on outbreak control for severe acute respiratory syndrome in Hong Kong. J Epidemiol Community Health.

[20] Liu X, Luo W-T, Li Y, Li C-N, Hong Z-S, Chen H-L (2020). Psychological status and behavior changes of the public during the COVID-19 epidemic in China. Infect Dis Poverty.

[21] Mazza C, Ricci E, Biondi S, Colasanti M, Ferracuti S, Napoli C (2020). A nationwide survey of psychological distress among Italian people during the COVID-19 pandemic: immediate psychological responses and associated factors. Int J Environ Res Public Health.

[22] Moccia L, Janiri D, Pepe M, Dattoli L, Molinaro M, De Martin V (2020). Affective temperament, attachment style, and the psychological impact of the COVID-19 outbreak: an early report on the Italian general population. Brain Behav Immun.

[23] MOH (2020a). COVID 19 dashboard: Saudi Arabia. https://covid19.moh.gov.sa/. Accessed 2nd July 2020

[24] MOH (2020b). MOH reports first case of coronavirus infection. https://www.moh.gov.sa/en/Ministry/MediaCenter/News/Pages/News-2020-03-02-002.aspx. Accessed 13 July 2020.

[25] NCMH (2020). National Center for Mental Health Promotion. http://ncmh.org.sa/index.php/pages/covid19. Accessed 13 July 2020.

[26] Qiu J, Shen B, Zhao M, Wang Z, Xie B, Xu Y (2020) A nationwide survey of psychological distress among Chinese people in the COVID-19 epidemic: implications and policy recommendations. General Psych 33(2) 10.1136/gpsych-2020-100213 PMC7061893 32215365

[27] Ridner SH (2004). Psychological distress: concept analysis. J Adv Nurs.

[28] Sareen J, Erickson J, Medved MI, Asmundson GJ, Enns MW, Stein M (2013). Risk factors for post-injury mental health problems. Depress Anxiety.

[29] SPA (2020). Saudi Arabia imposes 24-hour curfew https://www.spa.gov.sa/viewfullstory.php?lang=en&newsid=2071013. Accessed 10th April 2020.

[30] The Survey System sample size calculator (2020). https://www.surveysystem.com/sscalc.htm. Accessed 20 May 2020.

[31] Tiong XT, Abdullah NSS, Bujang MA, Ratnasingam S, Joon CK, Wee HL (2018). Validation of the Kessler’s psychological distress scale (K10 & K6) in a Malaysian population. ASEAN J Psychiatry.

[32] Yezli S, Khan A (2020). COVID-19 social distancing in the Kingdom of Saudi Arabia: Bold measures in the face of political, economic, social and religious challenges. Travel Med Infect Dis.

